# The Effectiveness of Instructor Course of Basic Maritime Emergency Care on Knowledge and Skill Among Laypersons: A Mixed Methods Design

**DOI:** 10.1155/nrp/5597849

**Published:** 2025-12-05

**Authors:** Soontorn Thassanee, Sarathep Prakit, Rumtiammak Siriporn, Wuthisuthimethawee Prasit

**Affiliations:** ^1^ Faculty of Nursing, Surat Thani Rajabhat University, Surat Thani, 84100, Thailand; ^2^ Ministry of Public Health, Nonthaburi, Thailand, moph.go.th; ^3^ Faculty of Nursing, Prince of Songkla University, Hat Yai, Thailand, psu.ac.th; ^4^ Department of Emergency Medicine, Faculty of Medicine, Prince of Songkla University, Hat Yai, Thailand, psu.ac.th

**Keywords:** emergency care, emergency medical services, health services, instructor training, maritime

## Abstract

**Introduction:**

Effectiveness of basic emergency care by bystanders is the cornerstone of ensuring maritime safety. This study aimed to assess the effectiveness of the BMEC‐TC program in enhancing both knowledge and practical skills related to maritime emergency care among layperson participants.

**Methods:**

A mixed‐methods design, integrating both quantitative and qualitative approaches to evaluate the program’s impact, was employed from March to October 2021. Pre‐ and post‐training assessments of knowledge and practical skills were utilized to measure learning outcomes. In addition, qualitative data were collected through group discussions to explore participants’ perceptions and experiences regarding the training program.

**Results:**

After participating in the BMEC‐TC program, both healthcare providers and laypersons demonstrated statistically significant improvements in knowledge scores (*p* < 0.001). Post‐test mean ranks were considerably higher than pretest ranks for both groups, indicating enhanced understanding. Skill scores after training were notably high for healthcare providers (*M* = 90.81, SD = 2.81) and laypersons (88.80, SD = 3.92), reflecting strong competency development. Practical assessments further confirmed substantial gains in clinical performance. Participants also reported high levels of engagement and widely acknowledged the training’s relevance and applicability to daily maritime emergencies. The findings highlighted four key elements: 1E: Empathy and Knowledge Exchange in Healthcare Training, 2E: Encouraging Knowledge Sharing and Active Learning through Real‐Life Experiences, 3E: Effective Communication for Practical Skills, and 4E: Ensuring Comprehensive Understanding to Prevent Misapplication.

**Conclusion:**

The BMEC‐TC program effectively improved knowledge and skills in maritime emergency care education within local communities. This study highlights the importance of an integrated training approach that combines cognitive learning with practical application.

## 1. Introduction

Effective emergency care in coastal areas is essential for marine and coastal safety [1–3]. According to specific marine environments, effective emergency care relied on layperson performance in basic emergency care and activation of emergency medical service and professional competencies in responding to acute illness and accident while traveling and at leisure [4, 5]. The WHO introduced the Community First Aid Responder curriculum, emphasizing essential first‐aid skills among laypeople to manage emergencies effectively, particularly within the “golden hour” immediately following incidents (when early care can minimize negative outcomes) [6]. There is a need to develop an instructor’s training curriculum and specific content guidance based on trainee backgrounds and community needs to facilitate standard training for laypersons in the community [7–9].

Training in maritime emergency care emphasizes outcome‐driven goals, empowering trainees to assess casualties’ needs and respond promptly until professional assistance arrives. International Maritime Organization standards mandate training in basic maritime personal survival skills, underscoring the importance of swift and efficient treatment to mitigate marine‐related injuries’ secondary complications [10, 11]. The study aimed to develop the Basic Maritime Emergency Care (BMEC) Training Course (TC) for instructors and to evaluate the effectiveness of facilitated learning experiences in enhancing layperson’s knowledge and skills.

## 2. Review of Literature and Conceptual Framework

The conceptual framework of this study was based on an andragogical theory that focuses on hands‐on, active learning methods that acknowledge the unique experiences and diverse learning styles of adult learners [12]. Sparrow et al. and Steinmayr et al. measured various indicators of learning outcomes, including procedural and declarative knowledge, as well as test performance, among others [13, 14]. Therefore, effective course design involves evaluating learning outcomes through rigorous assessments and reflecting on the teaching strategies implemented. This process ensures alignment between situational factors, learning goals, objectives, feedback methods, and teaching activities, with a strong focus on active learning to enhance both knowledge retention and learner engagement.

## 3. Study Aim and Hypothesis

This study aimed to evaluate the effectiveness of the BMEC‐TC for instructors enhancing both knowledge and skills of laypersons. The hypothesized was that the BMEC‐TC for instructors would significantly improve laypersons’ knowledge and practical skills, as evidenced by pre‐ and post‐training score comparisons, with a skill assessment score exceeding 80% indicating the course’s effectiveness.

## 4. Methods

### 4.1. Course Development

Nineteen experts were enrolled to review evidence and draft the BMEC‐TC requirements, with recruitment focused on individuals with deep knowledge of Thailand’s maritime safety system. The modified Delphi process was performed to summarize training programs for one‐day Maritime Emergency Management courses, outlining subjects and instructional strategies for instructor training. Experts affirmed the suitability of the BMEC‐TC for healthcare personnel to educate laypersons, highlighting its focus on self‐care, basic care for others, and activation of emergency medical services during shore‐based incidents, particularly addressing injuries and illnesses related to maritime environments, including water‐related trauma and marine‐specific medical conditions. The curriculum incorporates problem‐based learning grounded in andragogical theory, designing case scenarios that align with trainees’ prior experiences, enabling instructors to understand the learners’ needs and use techniques that promote real‐world application and practical learning outcomes.

### 4.2. Design

The study employed a mixed‐methods design, combining quantitative pre‐ and post‐training assessments (written tests and practical evaluations) with qualitative data from group discussions to assess the program’s impact on participants’ knowledge, skills, and perceptions. This study’s reporting followed The Strengthening the Reporting of Observational Studies in Epidemiology (STROBE) [15] and Standards for Reporting Qualitative Research (SRQR) [16], which provides a comprehensive framework for evaluating quantitative and qualitative data in mixed‐methods research.

### 4.3. Sample and Setting

Quotas for pilot training are accessible to a quota of “maritime zones of interest”, including the Gulf of Thailand, where the Southern Health Region Community offers 137 primary care, 16 secondary care, and 6 tertiary care services, as well as fire and rescue officers. The researchers used judgmental sampling to gather sufficient data from each subset to meet a quota. All 90 participants were selected for the healthcare provider program, consisting of professionals with experience delivering maritime emergency medical care. The group included 12 advanced emergency medical technicians, 8 emergency physicians, 2 fire and rescue officers, 8 paramedics, 5 public health officers (professional level), 25 emergency registered nurses, and 30 emergency registered nurses (professional level). The healthcare providers were recruited to serve as trainers and facilitators in the layperson training program. Their role included delivering theoretical instruction, supervising practical simulations, and evaluating participant performance based on predefined competencies related to maritime emergency care scenarios. For the evaluation course, the trainees comprised 90 participants for the layperson participants, determined using G Power (version 3.1.9.7) for a one‐group pre–post‐test study design [17]. An additional 100 participants were included to account for a 10% dropout rate. Trainees were selected from provinces bordering the sea, with random assignment facilitated by geographical considerations, including the presence of islands in southern Thailand. The inclusion criteria for trainees were as follows: participants had to be residents of coastal areas, have a role related to assisting individuals affected by maritime or shore‐based incidents, or not be currently involved but express willingness to volunteer in maritime emergency situations. All participants were required to be over 18 years of age. The exclusion criteria included individuals who were unable to attend the full duration of the training program or had significant physical or mental health conditions that could hinder participation in simulations or practical sessions.

### 4.4. Ethical Considerations

In accordance with the ethical standards of the Declaration of Helsinki, the participants of the study were thoroughly briefed on the research objectives and potential adverse outcomes associated with the intervention. Prior to commencement, written informed consent from participants was indispensable for their involvement in this study, and they were assured of their right to refuse to participate, or to subsequently withdraw, without giving a reason, and without this affecting their statutory rights. This research was approved by the relevant Institutional Review Board, Human Research Ethics Committee of the Faculty of Medicine, Prince of Songkla University (REC 64‐073‐20‐1).

### 4.5. Research Instruments

This study utilized two main instruments: a pre‐ and post‐training knowledge assessment and a practical skills evaluation through scenario‐based simulations. The knowledge assessment consisted of 20 multiple‐choice questions covering the Maritime Emergency Care System, equipment preparedness, and first‐aid decision‐making. The content validity of the knowledge assessment was confirmed with item‐objective congruence (IOC) values ranging from 0.6 to 1.0, and its reliability was verified with a KR‐20 coefficient of 0.87.

The practical skills tool was reviewed by a panel of three subject matter experts (SMEs), with IOC values ranging from 0.66 to 1.00, indicating strong agreement on its relevance. The instrument was also tested for reliability, yielding acceptable results and confirming its validity and reliability. Both pre‐ and postassessment results demonstrated significant improvements in participants’ knowledge and skills, confirming the program’s effectiveness in preparing healthcare workers for marine emergencies.

A one‐hour group interview was conducted at the end of the training program to collect qualitative data on participants’ experiences and suggestions for program enhancement. Participants provided written informed consent, acknowledging their voluntary participation and right to withdraw. Open‐ended questions were used to explore their views on various aspects of the BMEC‐TC program. Four questions were asked to encourage participants to freely express their perspectives, offering valuable insights into the program’s strengths and areas for improvement (Table 1). All questions were reviewed for content accuracy and edited by three experts, with two being experienced in spatial research and one being an expert in qualitative research. Nonverbal cues and participant engagement were also observed to enrich the responses. The interview, held in a roundtable format, fostered an open, collaborative discussion.

**Table 1:** Semistructured question guidelines for interviews.

Research Questions	R1: Could you share your experience with the training? What skills or knowledge did you gain?
	R2: Was the course content relevant and useful for your work or learning? Do you have suggestions for improvement?
	R3: Were there aspects of the training that could be improved or issues to address in future sessions?
	R4: What suggestions do you have to enhance the course for future participants?

### 4.6. Data Collection

This study was conducted between March and October 2021, following the approval of the research protocol. To evaluate participants’ knowledge and skills in maritime emergency care, both pre‐ and post‐training assessments were administered. These assessments included written tests to measure cognitive knowledge and practical skill evaluations to assess hands‐on competencies. In addition to these assessments, participant feedback was gathered through structured group discussions, which provided deeper insights into their perceptions of the training program. The combination of quantitative and qualitative data allowed for a comprehensive evaluation of the program’s effectiveness. The data collection process was meticulously designed to ensure a thorough analysis of both cognitive learning outcomes and practical skills acquisition, offering a well‐rounded understanding of the program’s impact. The high level of participant engagement and willingness to contribute to the discussions further enriched the data, fostering a collaborative and insightful dialogue that strengthened the study’s findings.

### 4.7. Data Analysis

Descriptive statistics summarized participants’ demographics. The Wilcoxon signed‐rank test was used to evaluate the pilot program’s effectiveness, with statistical significance set at *p* < 0.05. Participants’ scores were summarized using mean and standard deviation to assess trends and outliers. A one‐sample *t*‐test compared the mean score to the 80% passing threshold, while a proportion test determined the percentage of participants scoring above 80%. For participant reflections, content analysis was applied to focus group interview data, which were transcribed, validated, and translated by professionals. Transcriptions were reviewed for accuracy by the research team and an expert. Data were analyzed using Creswell’s six‐step method to identify recurring themes, which were reviewed and confirmed through member checking and triangulation to ensure rigor and validity [18].

## 5. Results

The effectiveness of the BMEC‐TC training program was evaluated by assessing participants’ knowledge and practical skills before and after the course. The cohort included both healthcare providers and laypersons, and their demographic and occupational characteristics were analyzed to understand how these factors might influence the learning outcomes (Table 2). The results demonstrated statistically significant improvements in both domains among healthcare providers. For the knowledge assessment, the Wilcoxon signed‐rank test showed no negative ranks and a positive rank sum of 4005.00, with a *Z* score of 8.206 and a *p* value of < 0.001. Additionally, the mean post‐test score for practical skills among healthcare providers was 90.81 (SD = 2.81) (Table 3).

**Table 2:** Characteristics of participants both healthcare providers and laypersons.

	Frequency	Percentage
Healthcare provider characteristics (*N* = 90)		
Gender		
Male	36	40.0
Female	54	60.0
Classification title		
Advanced emergency medical technician	12	13.33
Emergency physician	8	8.89
Fire and rescue officer	2	2.22
Paramedic	8	8.89
Public health officer (professional level)	5	5.56
Emergency registered nurses	25	27.78
Emergency registered nurse (professional level)	30	33.33
Layperson characteristics (*N* = 84)		
Gender		
Male	55	65.48
Female	29	34.52
Occupation		
Emergency medical technician (basic)	20	23.80
First responder	28	33.33
Park ranger	3	3.58
Local guide	24	28.58
Teacher	4	4.76
Tourist police	5	5.95

**Table 3:** Participants’ knowledge and skill scores before and after BMEC training.

	$\overset{¯}{x}$ (SD)	Mean rank	Sum of ranks	*Z*	*p* ^a^
Healthcare provider					
Knowledge					
Pretest		0.00	0.00	8.206	< 0.001^∗^
Post‐test		45.00	4005.00		
Skill	^∗∗^90.81 (2.81)				
Layperson (*N* = 84)					
Knowledge					
Pretest		15.00	60.00	7.597	< 0.001^∗^
Post‐test		42.86	3343.00		
Skill	^∗∗^88.80 (3.92)				

^a^Wilcoxon signed‐rank test, *p* < 0.05.

^∗^Statistically significant based on the Wilcoxon signed‐rank test (*p* < 0.05).

^∗∗^Descriptive statistics for skill scores; no inferential test was conducted for this variable.

Among laypersons, 100 participants were initially enrolled, but the final sample consisted of 84 due to participant withdrawals. The cohort included both healthcare providers and laypersons, and their demographic and occupational characteristics were analyzed to understand how these factors might influence the learning outcomes (Table 2). The results showed a significant improvement in both knowledge and skills, as evidenced by pre‐ and post‐test scores, with a *p* value less than < 0.001. Before the BMEC‐TC, participants’ knowledge was assessed through a pre‐test, yielding a mean rank of 15.00. The post‐test results showed a considerable improvement, with a mean rank of 42.86 and a *Z* score of 7.597, confirming the substantial difference. For skills, the laypersons scored an average of 88.80, demonstrating a high level of competence in practical emergency care. The assessment data, including mean scores, standard deviations, and statistical tests, confirmed the training’s success in enhancing both theoretical knowledge and practical skills (Table 3).

Participant feedback from reflective sessions was vital for refining the BMEC‐TC and improving the learning experience. A qualitative approach was used to gather insights from trainers and trainees after the program. In the final 60 min session, participants were divided into two groups for discussion. This revealed key themes, highlighting the program’s effectiveness and areas for improvement, providing a comprehensive understanding of its impact, benefits, and limitations. The trainer group included 6 registered nurses, 2 professional‐level nurses, 2 paramedics, and 2 emergency physicians, offering diverse professional perspectives. The trainee group comprised 10 participants: 2 emergency medical technicians (basic), 2 first responders, 2 tourist police officers, 2 park rangers, and 2 local guides. These sessions provided a platform for sharing experiences, insights, and suggestions related to the BMEC‐TC.

The findings highlighted four key elements: 1E: Empathy and Knowledge Exchange in Healthcare Training; 2E: Encouraging Knowledge Sharing and Active Learning through Real‐Life Experiences; 3E: Effective Communication for Practical Skills; and 4E: Ensuring Comprehensive Understanding to Prevent Misapplication.

### 5.1. Empathy and Knowledge Exchange in Healthcare Training

Before the training, all instructors took the time to inquire about and get to know the participants, which proved beneficial for the teaching process. By understanding their prior knowledge, work nature, and experience with basic care or local wisdom, the instructors tailored the learning experience accordingly. This approach fostered mutual understanding, encouraged open‐mindedness, reduced biases, and created an environment where participants felt comfortable sharing local knowledge.

Trainer no. 1, 2, 3, 5, 7, 11, and 12 emphasized the importance of using up‐to‐date empirical evidence and reliable references when presenting data to assist individuals affected by venomous and nonvenomous marine animals, especially those experiencing severe pain.

Trainer no. 7 shared that most trainees believed that lying on a fire‐heated bamboo bed frame could help relieve pain if stung by a stingray. However, based on previous research, hot water immersion has been found effective in alleviating pain, provided the water temperature is high enough to relieve discomfort but not so hot as to cause skin burns. A consistent temperature of 45°C for at least 20 min has been shown to reduce pain.

Trainer no. 11 shared, “During one of the training sessions, a trainee assisted a tourist on the beach who had been stung by a jellyfish by using vinegar from a bottle and spraying it as first aid before taking the victim to the hospital. This incident led to an introduction to the topic of jellyfish venom release mechanisms.”

### 5.2. Encouraging Knowledge Sharing and Active Learning Through Real‐Life Experiences

All instructors utilized teaching techniques that presented real‐life case examples from the local context.

Trainer no. 12 expressed that “the topic of first aid for victims of rip currents was discussed, during which one of the participants had the opportunity to speak with a survivor who shared their experience of swimming parallel to the shore to escape the rip current. He described how approach helped the survivors avoid being pulled further out to sea and allowed them to stay afloat while waiting for rescue by volunteer lifeguards. Such knowledge is critical for prevention and can be applied practically, such as by placing warning flags on beaches or sharing information with the community”.

Trainer no. 4 highlighted the traditional use of sea morning glory for jellyfish stings, while also introducing recent research recommending vinegar to rinse the affected area for 30 s to prevent toxin release. “I believe the instructor should present and share research references, encouraging volunteers, police officers, and community members to study together”. This collaborative approach fostered interest and enhanced understanding of the toxin‐release mechanism before discussing treatment methods.

In the trainee group discussion, participants highlighted the effectiveness of role‐playing, positive reinforcement, and constructive feedback. They emphasized the importance of using positive language in emergencies and ensuring a thorough understanding of the training content to prevent misunderstandings, which could lead to incorrect application and unintended harm in real‐life situations.

### 5.3. Effective Communication for Practical Skills

Layperson trainees also provided positive feedback, expressing appreciation for the course’s relevance and practicality. They noted that the curriculum expanded their knowledge and offered essential skills for real‐life situations.

Trainees no. 1, 3, 4, 6, and 9 highlighted new, easily implementable techniques, such as using positive language in emergencies to improve communication.

Trainee no. 18 appreciated the training format, noting that instructors used positive language, offered praise, and encouraged participation during group activities. “Thank you for your teaching. You explain each step clearly and don’t blame me for my mistakes (smiling). I feel comfortable asking questions and trying again.” There was also an emphasis on key actions to increase survival chances.

Trainee no. 20 noted, “Unlike previous trainings, here the instructors said ‘the patient could die if a mistake is made,’ which was a significant difference.”

### 5.4. Ensuring Comprehensive Understanding to Prevent Misapplication

However, some trainees from the layperson group highlighted limitations in this training. One participant stated loudly, “If the learners do not fully understand the content, it may lead to incorrect application, turning potential benefits into potential harm” (Trainee no. 18). This feedback highlights the crucial need to ensure that all participants thoroughly grasp the material to prevent misapplication in real‐world scenarios.

## 6. Discussion

The study demonstrated the BMEC‐TC program’s effectiveness in enhancing both knowledge and skills. The results demonstrated statistically significant improvements in both domains among healthcare providers. For the knowledge assessment, the Wilcoxon signed‐rank test showed no negative ranks and a positive rank sum of 4005.00, with a *Z* score of 8.206 and a *p* value of < 0.001, indicating a significant increase in knowledge following the training. Additionally, the mean post‐test score for practical skills among healthcare providers was 90.81 (SD = 2.81), reflecting high levels of competency attained through the program. These results provide a strong foundation for further exploration of the program’s potential impact on training healthcare providers, specifically instructors, in delivering maritime emergency care to laypersons. In Thailand, knowledge of maritime emergency care remains limited, with few training programs or publications available. The primary institutions involved, such as the Royal Thai Navy, offer training opportunities for healthcare professionals [19]. Consequently, initial knowledge scores among layperson participants are low, but they improve significantly after completing the training, and their scores on the skills test are higher. In local communities, maritime knowledge is often passed down through generations, relying on experiential learning rather than scientific validation by experts in the field [20]. The exchange of experiences and integration of local knowledge play a vital role in delivering emergency nurses in these areas [21].

Effective training requires not only the transfer of knowledge but also the creation of an environment where both instructors and learners engage in meaningful dialogue [22]. Utilizing in situ simulation as an educational strategy in the clinical environment is recommended [23]. The findings of this study align with previous research, which demonstrated that building confidence and practical competence through real‐world case studies can significantly enhance learning outcomes. By integrating these strategies, healthcare professionals can bridge the gap between theoretical knowledge and hands‐on practice, thereby improving overall clinical competency.

*Empathy and Knowledge Exchange in Healthcare Training* should focus on fostering mutual understanding between instructors and learners through compassionate data exchange, the application of scientific evidence, and the recognition of learners’ perspectives. This approach reflects the development of skills in patient care by integrating both relevant knowledge and personal experiences, ensuring that care is both evidence‐based and empathetic. Empathy is a crucial skill that begins with bachelor nursing education and continues through to nurse practitioners. It can be learned and enhanced through targeted training programs [24, 25]. The development of empathy is particularly important in health knowledge exchange, as it involves both understanding and relating to others’ beliefs, values, and differences, which are essential for providing comprehensive patient‐centered care, where ethnocultural empathy is crucial [26]. While nursing student programs demonstrated the highest empathy and those in intensive‐care nursing programs the lowest, there is a need for further development in integrating effective empathy training methods into nursing education curricula [25]. Additionally, knowledge transfer and exchange initiatives can facilitate the application of evidence‐based practices in healthcare, though more robust research is needed in this area [27].

While many healthcare institutions offer formal clinical skills training for nurses, it is often unclear whether the training itself or the clinical setting has a more significant influence on nursing practice [28]. *Encouraging Knowledge Sharing and Active Learning through Real-Life Experiences*, formal training is considered a valuable complement, rather than a substitute, for hands‐on learning in clinical environments [29]. This perspective is especially relevant when designing training programs for adult learners, where practical experience plays a key role in skill development. Previous research has explored methods for teachers to promote active learning and knowledge sharing, including workshops, collaboration, self‐study, and real‐life experiences [30, 31]. These studies conclude that integrating practical scenarios with interactive strategies, such as discussion, enhances student engagement and deepens understanding. However, the knowledge‐sharing method, particularly face‐to‐face communication, may impose limitations and create pressure within the learning environment [30].

Expanding upon earlier research findings, which highlight the effectiveness of interactive strategies and real‐life experiences in enhancing student engagement, this study similarly found that layperson trainees provided positive feedback, with many participants expressing appreciation for the course’s relevance and practicality. One technique frequently mentioned was the use of positive language during interactions, which trainees identified as a key strategy for improving communication, particularly in high‐pressure emergency situations, as demonstrated in *Effective Communication for Practical Skills*. Communication skills training for healthcare trainees, including the use of positive language, improves perceived performance and boosts confidence in managing patient interactions by fostering trust and reducing patient anxiety [32]. These insights demonstrate how well‐designed training programs provide immediate, tangible benefits for layperson learners, with trainees effectively integrating new techniques into daily practices, aligning with adult learning principles that emphasize relevance and immediate application.

While the BMEC‐TC program received positive feedback for its practical applications, a notable limitation was identified by some trainees, particularly from the local guide group. One participant highlighted that insufficient understanding of the content could lead to incorrect application, potentially causing harm rather than benefit. This emphasizes the need for all participants, regardless of prior knowledge, to achieve a comprehensive understanding to prevent misapplication. This issue reflects a broader challenge in adult education: the ongoing need to assess learners’ understanding. Although adult learners bring valuable real‐world experience, gaps in foundational knowledge may hinder their ability to correctly apply new concepts [33]. To improve the effectiveness of instructor training for layperson adult learners, key concepts should be reinforced through practical demonstrations, continuous assessments, and opportunities for clarification before real‐world application. Ensuring Comprehensive Understanding to Prevent Misapplication is critical. Adjusting the course design to incorporate more formative assessments, feedback, and hands‐on practice will help ensure full comprehension, thereby maximizing the training’s benefits and minimizing the risk of misapplication in critical maritime emergencies [34]. Furthermore, integrating research competence and assessing learners’ foundational knowledge before developing program content can enable educators to design skill‐building programs that effectively foster the essential skills and abilities required by adult learners [35]. By emphasizing both theoretical understanding and practical application, the program can better address the diverse needs of adult learners, ensuring that they are well‐equipped to apply their learning in real‐world settings.

## 7. Conclusions and Implications for Nursing Practice

This study highlights key implications for nursing practice: ensuring comprehensive understanding among participants to prevent skill misapplication, continuous assessment to address knowledge gaps, and adapting training for adult learners with practical experience but limited foundational knowledge. Effective communication, especially in high‐pressure situations, enhances trust and empathy. Integrating local knowledge with scientific evidence strengthens both knowledge retention and practical competence. The BMEC‐TC program provides valuable insights into improving maritime emergency care training, emphasizing comprehensive education, ongoing assessment, communication skills, and local knowledge integration for better preparedness in maritime emergencies.

## Disclosure

All authors critically reviewed, revised the manuscript, and approved the final version for publication. The corresponding author attests that all listed authors meet authorship criteria and that no others meeting the criteria have been omitted.

## Conflicts of Interest

The authors declare no conflicts of interest.

## Author Contributions

Soontorn Thassanee, Sarathep Prakit, and Wuthisuthimethawee Prasit were responsible for the conceptualization and design of this study. The data collection was conducted by Soontorn Thassanee, Sarathep Prakit, Rumtiammak Siriporn, and Wuthisuthimethawee Prasit. The data analysis and interpretation were carried out by Soontorn Thassanee, Sarathep Prakit, and Wuthisuthimethawee Prasit. Soontorn Thassanee drafted the initial manuscript. All authors take responsibility for the integrity of the data and the accuracy of the data analysis.

## Funding

The National Research Council of Thailand provided full funding for this study in 2021.

## Data Availability

The data supporting the findings of this study are available from the corresponding author upon reasonable request.
